# Facing Resistant Bacteria with Plant Essential Oils: Reviewing the Oregano Case

**DOI:** 10.3390/antibiotics11121777

**Published:** 2022-12-08

**Authors:** Jorge O. Fimbres-García, Marcela Flores-Sauceda, Elsa Daniela Othon-Díaz, Alfonso García-Galaz, Melvin R. Tapia-Rodríguez, Brenda A. Silva-Espinoza, Jesus F. Ayala-Zavala

**Affiliations:** 1 Centro de Investigación en Alimentación y Desarrollo, A.C, Carretera Gustavo Enrique Astiazarán Rosas 46, Hermosillo 83304, Mexico; 2 Departamento de Biotecnología y Ciencias Alimentarias, Instituto Tecnológico de Sonora, 5 de Febrero 818 sur, Col. Centro, Ciudad Obregón 85000, Mexico

**Keywords:** *Lippia graveolens*, carvacrol, antibacterial resistance, ESKAPE group, co-cultures, synergism

## Abstract

Antibiotic resistance is a serious global threat, and the misuse of antibiotics is considered its main cause. It is characterized by the expression of bacterial defense mechanisms, e.g., β-lactamases, expulsion pumps, and biofilm development. *Acinetobacter baumannii* and *Pseudomonas aeruginosa* are antibiotic-resistant species that cause high morbidity and mortality. Several alternatives are proposed to defeat antibiotic resistance, including antimicrobial peptides, bacteriophages, and plant compounds. Terpenes from different plant essential oils have proven antimicrobial action against pathogenic bacteria, and evidence is being generated about their effect against antibiotic-resistant species. That is the case for oregano essential oil (*Lippia graveolens*), whose antibacterial effect is widely attributed to carvacrol, its main component; however, minor constituents could have an important contribution. The analyzed evidence reveals that most antibacterial evaluations have been performed on single species; however, it is necessary to analyze their activity against multispecies systems. Hence, another alternative is using plant compounds to inactivate hydrolytic enzymes and biofilms to potentiate antibiotics’ effects. Despite the promising results of plant terpenes, more extensive and deep mechanistic studies are needed involving antibiotic-resistant multispecies to understand their full potential against this problem.

## 1. Introduction

Antibiotic resistance refers to the set of bacterial mechanisms expressed to avoid the effect of antibiotic drugs. According to the World Health Organization (WHO), the 700,000 annual deaths associated with multiresistant bacteria could increase to 10 million [1,2]. It is equally worrying that infections of this type require broader-spectrum and more expensive antibiotics, extending the patients’ agony and carrying a higher mortality risk [3,4]. The main causes triggering antibiotic resistance are excessive and inadequate prescriptions or poor infection control [5]. Similarly, intrinsic genetic mechanisms, such as efflux pumps, biofilm development, and hydrolytic enzymes, also cause this phenomenon [3]. Some antibiotics were effective for prolonged times, including vancomycin, released in 1958, and the first resistant bacterial strain appeared 30 years later. However, the first penicillin-resistant *Staphylococcus aureus* strain appeared one year after the antibiotic release [6]. Therefore, antibiotic resistance is a serious and multidisciplinary problem that requires urgent attention.

The upward trend of antibiotic resistance and its impact has been observed in the USA for the Extended-Spectrum Beta-Lactamase (BLEE)-producing *Enterobacteriaceae*, with 65,500 more cases of hospitalized patients in 2019 compared to 2021 [6]. β-lactamase are hydrolytic enzymes that degrade β-lactam antibiotics, and these predominate due to their abundant distribution and high hydrolysis capacities, such as metallo-β-lactamases and oxacillinases. Therefore, the searched alternatives and adjuvant treatments include new effective molecules, vaccines, antibody and immunomodulatory therapies, antibiotic potentiators, and antivirulence agents [4]. Specifically, among the explored treatments are bacteriophages, probiotics, peptides, and secondary plant metabolites; this review is focused on the potential of terpenes found in plant essential oils.

Rubio-Ortega et al. [7] studied the minimum inhibitory (MIC) and bactericidal concentration (MBC) of *Thymus vulgaris* and *Lippia graveolens* against *Salmonella enterica* subsp. *enterica* and serovar Typhimurium, reporting their efficacy in a range of 0.5–1 mg/mL. On the other hand, El-Said et al. [8] evidenced the antibacterial efficacy of *Lippia pubescens*, presenting inhibition halos of 10 to 24 mm for *E. faecalis, S. epidermidis*. *A. baumannii*, and *S.* Typhimurium. Rubio-Ortega et al. [7] also showed that *T. vulgaris* and *L. graveolens* caused inhibition halos from 20.7 to 60 mm *Salmonella enterica* subsp. *enterica* and serovar Typhimurium. Deeper studies conducted by Reyes-Jurado et al. [9] evidence that *Lippia berlandieri* oil at 600 mg/L and 250 mg/L reduced the biofilm formation of *P. aeruginosa* and *S.* Typhimurium, respectively. Meanwhile, Karumathil et al. [10] analyzed resistance-gene regulation in *A. baumannii* strains using trans-cinnamaldehyde and observed a reduced expression of *bla*P genes, coding for β-lactamase. Despite the last two listed exceptions, most reviewed evidence only determined bacterial sensitivity to the tested treatments by agar diffusion and provided MIC and MBC values, lacking mechanistic approaches.

Combining plant compounds with antibiotics has shown promising synergistic effects against bacterial resistance. Guo et al. [11] observed that capsaicin decreased the MIC of colistin below 2 mg/mL against colistin-resistant *A. baumannii* strains. In the same sense, Amaral et al. [12] found a 16-fold reduction in the MIC value of polymyxin B combined with oregano essential oil against the same pathogen. The carvacrol–meropenem combination tested by Odabaş-Köse [13] inhibited the growth of carbapenem-resistant *Klebsiella pneumoniae*. These promising results open the possibility of exploring the efficacy of more combinations with different terpene–antibiotic molecules against other pathogenic species; in addition, the mode of action of each constituent must be considered. Most reviewed articles were focused on *Origanum vulgare*, and other oregano species with similar composition remain underestimated, such as *L. graveolens*.

More complex bacterial ecosystems need to be considered when testing antibacterial agents. Chan et al. [14] introduced the coculture technique and observed the up-regulation of 909 genes in *K. pneumoniae* and 388 genes in *A. baumannii*, including antibiotic-resistance-related genes. However, this study did not challenge the coculture system with antibacterial agents. These experimental conditions would allow for knowing the antibacterial activity of selected molecules and the effect of inter-species interactions against co-infection processes. Therefore, this review discusses the potential benefit of combining essential oil terpenes with antibiotics to attack resistant multispecies complexes.

## 2. Antibiotic Bacterial Resistance: A Serious and Multidisciplinary Problem

Antibiotics are the main treatment against bacterial infections in animals and plants [15]. As explained before, efficacy loss is known as antibiotic resistance; this manifestation occurs when bacteria mutate in response to excess and sublethal doses of these drugs [1,16,17,18]. Antibiotic resistance is usually classified into intrinsic and acquired resistance. The first one is constitutive of each species, which means that each generation will present it. In contrast, the acquired mechanism occurs when bacteria obtain resistance genes through conjugation, transformation, transduction, and transposition [2,3]. Regardless of the type of resistance, both cause this problem.

According to The United Nations, bacterial resistance is “one of the major health threats (…) endangering (…) human development” [1]. This perception becomes relevant due to the high mortality rates associated with multidrug-resistant infections. Septicemia is an example, as 30% of newborns who suffer from it die [3,19]. On the other hand, according to the CDC [20], the United States reported 2 million bacterial infections caused by resistant species and 23,000 deaths in 2013; in 2019, the incidence increased to 2,500,000 infected persons and 35,000 deaths. The WHO reports that the pathogens known as ESKAPE, formed by *Enterococcus faecium, S. aureus, Klebsiella pneumoniae, A. baumannii, P. aeruginosa* and *Enterobacter* spp. are the cause of HAIs (Healthcare-Associated Infections), accounting for up to 67% of all infections. As a result, the ESKAPE group is considered a priority when deciding clinical treatments (Table 1) [1,4,21].

For example, in the United States, hospital-acquired *E. faecium* infection rates were 14% of the total infections reported between 2011 and 2014, while *A. baumannii* presented 2% and up to 4% in Asia [22]. A database analysis in the United States conducted by Marturano and Lowery in 2019 [23] showed that ESKAPE pathogens represented 42.2% of total bloodstream infections. These results coincide with the Department of Plastic and Burn Surgery of the Affiliated Hospital of Southwest Medical University in Luzhou, China, informing us that 47.5% of bacterial isolates from burn patients corresponded to the ESKAPE group [24]. Furthermore, 55% of these isolates were identified as multidrug resistant (MDR). In Mexico, Sosa-Hernández et al. in 2019 [25] estimated that 42.2% of HAIs in a hospital during 2013–2017 were attributed to the ESKAPE group. In turn, in an intensive-care unit in Monterrey, Nuevo Leon, Mexico, in 2012, this group accounted for 64% of isolates and 86.2% of *A. baumannii* and 28.9% of *P. aeruginosa* were MDR [26]. This review selected *A. baumannii* and *P. aeruginosa* to represent the ESKAPE group impact; these two species are among the greatest concerns due to their pathogenesis and high rates of resistance, morbidity, and circulation in hospital settings.

The epidemiology of bacterial resistance must include constant monitoring of the circulant pathotypes and antibiotic-resistant profiles. Therefore, in most countries, research must be expanded on monitoring and characterizing bacterial antibiotic resistance. This action is relevant because it would allow for a better understanding of this global challenge [27]. This situation represents a challenge since a correct antibiotic-resistance characterization is needed, but it is a long process. However, a proper approach to this phenomenon allows us to understand its behavioral tendency in different environments. Therefore, since this review intends to focus on these bacterial species as representative of MDR species, their epidemiological development is discussed more specifically.

The genus *Acinetobacter* is defined as “short pleomorphic coccobacilli that are Gram-negative, strict aerobic, catalase positive, oxidase negative, non-fermenting and immotile” [28]. Some species of this genus can be found in different environments; however, *A. baumannii* is mainly associated with hospital settings [28,29]. One million cases of infection are attributed to this species each year [30], with mortality rates ranging from 23 to 68% for HAI and up to 64% for community-acquired infections.

On the other hand, pathologies attributed to *A. baumannii* can be found in the respiratory tract, bloodstream, urinary tract, and wounds [10]. In Lebanon, Kanafani et al. [31] evidenced that respiratory tract infections caused by this pathogen accounted for 53.1% of total cases. It was found that 68.3% of *A. baumannii* isolates from ventilator-associated pneumonia (VAP) corresponded to Extensive Drug Resistance (XDR). In comparison, 13.3% was MDR and 18.3% Pan-drug resistant (PDR) [32]. Furthermore, an international meta-analysis revealed that *A. baumannii* MDR prevalence is related to hospital-acquired pneumonia and represents almost 80% of the analyzed cases [28]. The prevalence of antibiotic-resistant *A. baumannii* strains indicates the relevance of designing strategies for controlling their virulence and dispersion.

*Pseudomonas aeruginosa* is a Gram-negative, rod-shaped, non-fermenting, motile, oxidase-positive, facultative aerobic bacillus, considered a non-fastidious microorganism in terms of its growing conditions. It is ubiquitous and persistent in water and soil, although it is mostly associated with predominance in clinical settings [4,33]. According to statistics in the United States of America, 13–19% of nosocomial infections are attributed to *P. aeruginosa*, mainly affecting ICUs (Intensive-Care Units), and can represent up to 23% of all cases. Its presence is manifested in clinical conditions, such as pneumonia, skin, ear, eye, urinary tract, and bloodstream infections, the most frequent in the respiratory tract [4,33,34]. Up to 22% of all HAIs is described by healthcare-associated pneumonia and VAP, where *P. aeruginosa* accounts for 10–20% of isolates, with an estimated mortality of 32–42.8% [34]. These infection and mortality levels may be associated partly with the rapid development of resistance to therapeutic agents. According to the National Healthcare Safety Network, during 2015–2017, 26.3% of *P. aeruginosa* isolates in ICU patients with possible pneumonia had carbapenem resistance [34]. Therefore, it is urgent to monitor the development of this phenomenon.

### 2.1. Consequences of Bacterial Resistance

The costs associated with bacterial-resistant infections could reach USD 100 trillion globally [1,2]. It could also reduce Gross Domestic Product by 2–5% in some countries, generating 24 million more people in extreme poverty by 2030 [1,35]. In addition, drug resistance can extend hospitalization periods, involving a greater risk of death, decreasing the quality of life, and requiring the use of drugs with a wider spectrum and higher cost [3,4].

The attack of antibiotic-resistant bacterial infections increases vulnerability in some daily clinical practices, such as surgery, organ transplantation, or chemotherapy [16]. Unfortunately, these consequences would be accentuated in the most vulnerable social strata, elderly, immunocompromised, and low-income populations [19]. Finally, it should be kept in mind that the consequences of this phenomenon cannot be solved only with the creation of new drugs but with worldwide political and social changes.

### 2.2. Factors Causing Bacterial Resistance to Antibiotics

The development of antibiotic resistance has been conventionally attributed to two main mechanisms. The first mechanism involves the genetic responses triggered by the antibiotic challenge. The second mechanism considers a more complex system formed by environmental factors [36]. The genetic mechanisms of antibiotic resistance can be summarized in three general processes: regulation in antibiotic concentration, enzymatic antibiotic processing, and alteration of its target site [37]. More specifically, Serra-Valdés [3] lists the following mechanisms as the main ones: (i) Increased function of efflux pumps to release the antibiotic from bacterial cells. (ii) Hydrolytic enzymes to inactivate the antibiotic. (iii) Modifying PBP (penicillin-binding protein) to avoid antibiotic recognition. (iv) Decreased cell membrane permeability and biofilm development to block antibiotic access. (v) Overexpression of the target site. It is important to mention that all these responses can act simultaneously, taking a more complex picture.

In some ESKAPE group members, resistance mechanisms have a certain specificity. Resistance in *A. baumannii* is reflected in reduced membrane permeability, altered antibiotic target sites, and increased efflux pump function [38]. For example, overexpression of AdeABC efflux pumps, OmpA, CarO porins, and antibiotic hydrolysis provides resistance to *A. baumanii* against β-lactams. β-lactamases can be classified into four categories, A, B, C and D; *A. baumannii* has all four types of these enzymes, VIM, IMP, NDM, and ADC inherent to the species and OXA type. OXA-23 and OXA-51 are mainly involved in carbapenem resistance [37]. Resistance in *P. aeruginosa* has some variations when compared to *A. baumannii*; it used the expression of efflux pumps, Amp-C, BLEE, and metallo-β-lactamases, and modifications in PBP, OprD, and OprH porins [4]. Consequently, resistance systems and pathogenicity factors determine bacterial infections’ course and severity; these differences could be considered to direct antivirulence treatments at gene and protein levels.

The role played by environmental and consumer-behavior conditions in antibiotic resistance is of great relevance. The CDC [5] states the following list as the main events driving this phenomenon: (i) over-prescription of antibiotics; (ii) patients not following prescriptions; (iii) unnecessary use in agriculture; (iv) poor infection control in hospitals and clinical settings; (v) poor hygiene and sanitation practices in infected animals and plants; and (vi) lack of rapid laboratory tests for antibiotic-resistance detection. In addition, other factors, such as lack of access to clean water, poor access to quality drugs, vaccines, diagnostic tools, lack of awareness and knowledge, non-compliance with legislation, and indifference of pharmaceutical companies, also contribute to this problem. Hence, it is important to consider all these issues as priorities when taking action [4,36].

## 3. Multispecies Challenge Involved in Antimicrobial Testing

A simple way to characterize sensitivity to antibacterial agents is the addition of different concentrations in culture media. This practice has been routinely performed for some time; however, microbial growth in nature may not occur as single species. For this reason, the knowledge of the multispecies interaction and the treatment response against these systems is limited. An alternative approach to cope with these variations is the so-called coculture, which aims to mimic the natural relationship between the study microorganisms [39]. Multispecies coculture can provide valuable information on the ecological behavior of pathogenic and beneficial species.

The first part of the multispecies challenge is to define the microorganisms to be combined [40]. In addition, it is important to understand the bacterial ecology in terms of inter-, intra-species, and environmental interactions that could favor the development and dominance of a given response against the treatment. The appropriate growing conditions must be chosen, such as a nutrient source and temperature, as this can determine bacterial behavior [41]. Another consideration is the treatment doses; each species can respond differently. Some scenarios could contemplate the treatment of pathogen-beneficial microbiota combination, expecting pathogen inhibition without affecting beneficial bacteria. In addition, the designed antimicrobial system must consider the possible practical uses.

Cocultures of multispecies bacteria are relevant because they allow for “the study of interspecies interactions, development of multispecies biofilms, ecology dynamics and construction of synthetic communities with specific functionalities” [40]. However, coculture research can show interactions between several microorganisms involved in an infection, and multiresistant pathogens are the ones of greatest interest [14]. Polymicrobial infections are usually more difficult to treat and multispecies can also develop biofilms, increasing their resistance [42,43]. Therefore, coculture could provide useful information in resolving infection and resistance problems.

Khan et al. [41] studied the interactions between *Halomonas* sp. HL-48 and *Marinobacter* sp. HL-58, when using different carbon sources and changed their molecular phenotypes. Specifically, it was observed that using glucose as a carbon source showed a competitive relationship between these two bacteria. Compared to the axenic culture, *Halomonas* HL-48 only increased its growth rate by 6%, while *Marinobacter* HL-58 decreased its growth rate by 20%. On the other hand, if the medium contained only xylose, which *Marinobacter* does not metabolize, the relationship became commensal. *Marinobacter* HL-58 in the coculture had good growth compared to the axenic culture; this was attributed to the secondary metabolites produced by *Halomonas* HL-48. Chan et al. [14] showed that the coculture between *K. pneumoniae* and *A. baumannii*, both MDR, led to the up-regulation of 909 and 388 genes in each species, respectively. They also described that some of these genes were related to antibiotic resistance. In addition, Maglangit et al. [39] reported that the coculture of *Streptomyces* sp.MA37 with *Pseudomonas* sp. activated the production of the pyrroloindolocarbazole alkaloid toxin, BE-13793C; however, this response was not detected in monocultures.

Antibacterial evaluations in coculture models are often scarce, especially using plant compounds. The study conducted by Barraza and Whiteley [43] reported that in coculture between *P. aeruginosa* and *Staphylococcus aureus*, 2-heptyl-4-hydroxyquinoline N-oxide was produced, which affected the physiology of *S. aureus* and increased its susceptibility to certain antimicrobials. Tamanai-Shacoori et al. [44] reported that combining silver zeolite with phenolic extracts of *Ascophyllum nodosum* inhibited the biofilm formed by *Streptococcus gordonii*. Still, a biofilm reduction was observed via the coculture of *Porphyromonas gingivalis* and *S. gordonii*. However, treating biofilms formed by monocultures of *S. gordonii* with the combination of antibacterial agents was more effective than using Ag-zeolite alone. In another study, Boulanger et al. [45] evaluated the effect of tomatidine (plant alkaloid) on the coculture of *P. aeruginosa* and *S. aureus*. It was observed that tomatidine did not exhibit a significant antibacterial effect against *S. aureus* monoculture. Otherwise, testing the compound under coculture conditions achieved a bactericidal effect on *S. aureus,* in addition to the effect caused by a *P. aeruginosa* by-product.

Clinical reports of co-infection have been evidenced among some members of the ESKAPE group. Only 8.33% of patients simultaneously presented a co-infection with *P. aeruginosa* and *A. baumannii* [46]. Most studies of multispecies interactions have focused on analyzing the production of secondary metabolites and species interaction. More attention should be paid to evaluating changes in cellular densities, biofilm formation, and antibiotic resistance at gene and protein levels. In addition, novel antibiotic substances could be tested against multispecies systems to prove their efficacy against this more complex system.

## 4. Efficacy Loss of Conventional Antibiotics and Proposed Solutions

Alternative techniques to conventional antibiotics include bacteriophages and endolysins, antimicrobial peptides, siderophores, photodynamic therapy, vaccines, nanoparticles, monoclonal antibodies, and active compounds from plants and fungi [4]. As a result, these alternatives have been proposed to solve this phenomenon, at least partially. Phage therapy treatment is directed to a given bacterial species and can be combined with conventional antibiotics [4]. In addition, they represent an effective alternative due to their zero toxicity, low cost, and specific response according to the microbiome [47]. Endolysins are enzymes produced by phages during their replication, and their action is exerted on the bacterial cell wall; therefore, they mainly affect Gram-positive bacteria. However, endolysins with specialized lytic domains can interact with cell membranes and affect Gram-negative bacteria [48]. However, both phages and endolysins still present certain restrictions for their human use.

Two other alternatives are antimicrobial peptides and nanoparticles. These peptides can be natural or synthetic, consisting of 11 to 50 amino acids, positively charged and amphipathic, affecting bacterial cell walls, proteins, and nucleic acids [49]. They can exert bactericidal and immune regulatory effects, splitting into bacteriocins or host defense peptides [50]. Despite these findings, there is still a lack of knowledge regarding interactions with target and host cells. On the other hand, nanoparticles are composed of organic or inorganic materials (such as metals) at a nanoscale, whose mode of action is interacting with bacterial cell walls and membranes, causing damage, free radical induction, enzymatic inhibition, and down-gene regulation [51,52]. Despite the very promising results of these alternatives, they may not be the most viable option, mainly due to unproven safety.

Plant compounds have been used as a source of human drugs since ancient times, and they have offered excellent alternatives against different illnesses, including bacterial infections. Plant molecules can also be considered alternatives against antibiotic-resistant bacteria, especially those compounds with low toxicity and viable to be obtained or synthesized [52]. The antibacterial action of some plant extracts is related to their composition, which is effective against antibiotic-resistant strains [53]. These phytochemicals can belong to various classes of organic compounds, such as alkaloids, phenols, flavonoids, and terpenes [52,53]. These have recently gained great popularity due to their antibacterial effectiveness, and they will be discussed in more detail.

Essential oils are a mixture of natural products, such as phenylpropanoids, alkaloids, fatty acid derivatives, coumarins, and terpenes, which grant different bioactive properties. [52,54,55]. Although each compound contributes to the given essential oil bioactivity, it has been found that terpenes are the main constituents in most essential oils and are associated with their antibacterial activity [54]. Therefore, this section discusses the scientific evidence supporting the viability of using plant compounds, with greater emphasis on terpenes, against antibiotic-resistant bacteria (Table 2).

Kumara et al. [56] reported that cinnamon, clove, capsicum, thyme, oregano, rosemary, and silver yarrow essential oils have strong antibacterial activity against *P. aeruginosa*. Eucalyptol and camphor were the active terpenes related to the silver yarrow oil. In this context, Tiwari et al. [57] reviewed the activity of *Lythrum salicaria* plant extracts against *P. aeruginosa* and *A. baumannii*. Similarly, El-Said et al. [8] evaluated the effect of 15 essential oils via agar diffusion on *Salmonella enterica* strains. Among all the plants, *T. vulgaris* and *L. graveolens* showed the highest efficacy with inhibition halos >20 mm. In addition, their MIC and MBC were determined, indicating that the essential oil of *L. graveolens* was the most effective, with a value of 0.5 mg/mL for both concentrations. In comparison, *T. vulgaris* had an MIC and MBC of 1 mg/mL.

Bernal-Mercado et al. [58] studied the antibacterial effect of vanillic, protocatechuic, and catechin acids against uropathogenic *E. coli* (UPEC). It was observed that exposing these bacteria to concentrations of 0.0010 mg/mL protocatechuic acid, 0.001 mg/mL vanillic acid, and 0.0020 mg/mL catechin affected biofilm formation. However, combining these three compounds in doses of 0.00024 mg/mL + 0.00012 mg/mL + 0.000014 mg/mL, respectively, was more effective against biofilm development. In addition, Cruz-Valenzuela et al. [59] determined that the aqueous extract of *Punica granatum* L. inhibited the growth of *Listeria monocytogenes* and *S.* Typhimurium at concentrations from 10 to 30 mg/mL (Ambrosio et al. [60]). For *E. coli.* it was determined that the major terpene in the whole oil was limonene; however, it was not found in the most effective fraction (4); this fraction had an MIC of 3.70 mg/mL against *E. coli.* This research established that the efficacy against bacterial pathogens observed in the whole essential oil (1.85 mg/mL) is greater than that found in fraction 4 and can be attributed mostly to mixture of the components, beyond its major compound. Guimarães et al. [61] examined the terpineol and thymol activities against *Staphylococcus aureus*, showing MICs of 0.03 and 0.007 mg/mL and CMBs of 0.12 mg/mL. In addition, Siddique et al. [62] examined *Zingiber montanum* against methicillin-resistant *S. aureus* (MRSA). (E)-8(17),12-labdadiene-15,16-dial *and* zerumbol were most effective among eight isolated terpenes, inhibiting the growth of MRSA at concentrations between 0.032 and 0.128 mg/mL.

Sakkas et al. [63] evaluated five essential oils on MDR isolates, and the most effective treatments were *Thymus capitatus* and *Melaleuca alternifolia*, presenting the lowest MICs of 0.25% and 0.12% v/v, respectively. In addition, the antibacterial potential of these oils was reported against *P. aeruginosa* (MIC 2–4 % *v/v*) and *A. baumannii* (MIC 0.25–0.37% *v/v*). In addition, El-Said et al. [8] examined essential oils from *L. pubescens*, *P. incisa* subsp. *candolleana and J. procera* against 13 Gram-positive and Gram-negative strains. Then, favorable results were obtained by adding 200 µg *L. pubescens* oil, observing inhibition halos of 10 to 15 mm against *A. baumannii*, *S.* Typhimurium, *Shigella sonnei, E. faecalis*, and *S. epidermidis*, in comparison with the halos produced by ciprofloxacin, vancomycin, and amikacin (9–30 mm); the oil effect can be attributed to its carvacrol content.

Jan et al. [64] demonstrated the antibacterial activity of wild and cultivated *O. vulgare* against *A. baumannii* and *P. aeruginosa*, reporting MIC values of 13.78 and 62.5 µL/mL, and attributed this effect to carvacrol and thymol. Coccimiglio et al. [65] reported MICs of 6.3–25 μg/mL of ethanolic extracts of the same plants against clinical isolates of *P. aeruginosa*. Regarding *L. graveolens*, Hernandez et al. [66] reported inhibition zones between 24.85 and 29.23 mm against *Salmonella* sp. and between 24.82 and 24.95 mm in *Pseudomonas fragi*. On the other hand, Bautista-Hernández et al. [67] reviewed the antibacterial action of *L. berlandieri* applied as vapor against *E. coli*, finding an MIC of 4 μg/mL. All the revised studies showed the antibacterial activity of plant compounds expressing MIC, MBC, or agar diffusion. However, further information about antivirulence mechanisms and their contribution against β-lactamases, efflux pumps, and biofilms is needed.

In contrast, other studies tried to be more consistent and aimed to demonstrate the site of action of these compounds in the bacterial cell (Table 2). In the case of essential oils, it is shown that they affect the bacteria membrane and cell wall, causing alterations and increasing permeability [57,65]. The proposed action of carvacrol and oregano essential oil influenced the membrane lipid composition, which induced ATP loss and inhibited toxin secretion [56]. Similarly, Montagu et al. [68] demonstrated that the individual and combined antibacterial activity of carvacrol and cinnamaldehyde caused the overexpression of stress-response genes (*groES, groEL,* and *dnaK*) in exposed *A. baumannii*. Mesquita et al. [69] evaluated *Lippia alba* essential oil antimicrobial activity. In that study, MIC and MBC (0.5–1 mg/mL) were determined; additionally, 1 mg/mL inhibited the biofilm development of *S. aureus*.

Moo et al. [70] determined the mechanism of action of eucalyptol against carbapenemase-producing *K. pneumoniae.* They established an MIC of 28.83 mg/mL. While at a concentration of 14.42 mg/mL, it caused damage to the outer membrane, induced cell lysis, and leakage of intracellular material, compared to untreated cells. Similarly, Reyes-Jurado et al. [9] reported the *L. berlandieri* anti-biofilm potential against *P. aeruginosa* and *Salmonella* Typhimurium in stainless-steel surfaces, obtaining concentrations of 600 mg/L and 250 mg/L, respectively. In this context, Karumathil et al. [10] detailed trans-Cinnamaldehyde and eugenol interactions with bacterial membranes, causing permeability and down-expression of genes related to efflux pumps. Trans-Cinnamaldehyde at 4 mM concentration suppressed the AdeABC efflux pump proteins. However, the outer membrane did not change permeability upon bacteria exposure to both compounds. On the other hand, combinations of trans-cinnamaldehyde or eugenol with antibiotics decreased bacterial resistance, causing a down-regulation of 3- to 14-fold in resistance genes, such as *blaP, mdrp, adeA*, and *adeB*. On the contrary, it is observed that antibacterial activities are generally tested against axenic cultures, as evidenced above. This caused a lack of knowledge about the expected efficacy against multispecies.

Finally, an alternative has gained great relevance is the synergistic combination of plant compounds and antibiotics. Guo et al. [11] found synergistic fractional inhibition concentration indexes (FICI) between 0.03 and 0.06 for capsaicin–colistin mixtures. For their part, Amaral et al. [12] tested the combination of *O. vulgare* essential oil with polymyxin B. They reported synergistic FICI values from 0.18 to 0.37, with concentrations of 0.015 µg/mL and 0.43 mg/mL of the antibiotic and oil, respectively, while Odabaş-Köse [13] reported an FICI of 0.5 when combining carvacrol and meropenem at varying concentrations of 32–128 µg/mL. Guo et al. [11] observed that the synergistic mixture of capsaicin-colistin up-regulated 271 genes, while 327 genes decreased their expression in *A. baumannii,* including some related to antibiotic resistance. However, the pos-transductional effect of the treatments against antibiotic resistance and virulence proteins was not evaluated.

Despite these last revised pieces of evidence, few studies contemplated mechanistic studies to elucidate molecular target points to decrease virulence and bacterial resistance. In addition, the antimicrobial challenges must consider simulations of extreme conditions to demonstrate the efficacy of individual and combined antimicrobial agents. Based on the above, the research question in this review arises: What is the effect of combining *L. graveolens* terpenes with antibiotics on the viability and virulence of resistant bacteria at single and multispecies levels?

**Table 2:** Previous studies on the antibacterial activity of essential oils, their constituents, and antibiotics.

Compounds	Species	Antibacterial Evaluation	Results	References
Carvacrol Cinnamaldehyde	*A. baumannii*	Gene expression	MIC Carvacrol-Cinnamaldehyde: 0.16 mg/mL	[68]
			*groES*, *groEL, dnaK*: overexpression 3.9–5.1-fold.	
			*clp B*, *kat E*: overexpression 26-fold, 20-fold	
EO *T. vulgaris* EO *L. graveolens*	*S. enterica*	Inhibition zone MIC/MBC	>20 mm 0.5–1 mg/mL	[7]
EO *Lippia alba* Citral	*Staphylococcus aureus*	Inhibition of biofilm	EO *L. alba:* 1 mg/mL	[69]
		data	Citral: 0.5 mg/mL	
EO *L. berlandieri*	*P. aeruginosa*	Inhibition of biofilm	250–600 mg/L	[9]
	*Salmonella Typhimurium*			
trans-Cinnamaldehyde Eugenol Antibiotics β-lactams and monobactams.	*A. baumannii*	Efflux pumps and resistance gene expression	MIC: 4 mM Supression of AdeABC efflux pump. Genes *adeA* and *adeB*: downregulated 3–14-fold Genes *blaP, mdrp*: downregulated 3-fold	[10]
EO *Lavandula pubescens* Carvacrol	*Acinetobacter baumannii, Salmonella typhimurium, Shigella sonnei, Enterococcus faecalis y Staphylococcus epidermidis*	MIC MBC Inhibition zone	MIC EO: 78–312 µg/mL MBC EO: 156–625 µg/mL MIC carvacrol: 250–500 µg/mL MBC carvacrol: 500–1000 µg/mL Inhibition zone 12–24 mm	[8]
EO *Ocimum basilicum* EO *Thymus capitatus* EO *Melaleuca alternifolia* EO *Thymus vulgaris*	*A. baumannii, E. coli, K. pneumoniae, P. aeruginosa*	MIC MBC	*Melaleuca alternifolia:* 0.12–1.50 (%*v/v*) *Thymus capitatus* y *Thymus vulgaris:* 0.5–>4 (%*v/v*) *Ocimum basilicum:* >4 (%*v/v*)	[63]
Capsaicin Colistin	*A. baumannii*	Synergism Gene regulation	FICI = 0.03–0.06 Increase 271 genes Decrease 327 genes Inhibition efflux pumps	[11]
Polymyxin B EO *Origanum vulgare*	*A. baumannii*	MIC Synergism	MIC = 1.75–3.50 mg/mL FICI = 0.18–0.37	[12]
Carvacrol Meropenem	*K. pneumoniae*	MIC Synergism	MIC = 32–128 µg/mL FICI = 0.5	[13]
EO *Origanum vulgare*	*A. baumannii* *P. aeruginosa*	MIC	13.78–62.5 µL/mL	[64]
Extract of *Origanum vulgare*	*P. aeruginosa*	MIC	6.3–25 µg/mL	[65]
EO *L. graveolens*	*P. fragi* *Salmonella* sp.	Inhibition zone	24.82–24.95 mm 24.85–29.23 mm	[66]

EO: Essential Oil.

## 5. Antibacterial Capacity of *L. graveolens* and *O. vulgare*

*L. graveolens* and *O. vulgare* species are called “oregano”, each exhibiting certain chemical particularities. Oregano is a plant usually grouped into four distinct families: *Lamiaceae, Verbenaceae, Asteraceae,* and *Fabaceae* [71]. While *O. vulgare* belongs to the family *Lamiaceae* and its main constituent is thymol, *L. graveolens* belongs to the family *Verbenaceae* and the main compound is carvacrol [71,72]. According to their nature, the bioactive compounds of oregano are usually divided into volatile (mainly terpenes) and non-volatile compounds (flavonoids or phenolic acids) [72]. The main terpenes found in oregano essential oil are carvacrol and thymol, followed by γ-terpinene, p-cymene, terpinen-4-ol, linalool, β-myrcene, hydrated trans-sabinene, and β-caryophyllene. [55].

Thymol and carvacrol are found in higher percentages than other *L. graveolens* constituents; evidence reflects that these are the most directly involved in the observed antibacterial efficacy. However, both terpenes possess a variety of important biological properties. Thymol can inhibit pro-inflammatory molecules, neutralize free radicals, and be antibacterial, antifungal, antiproliferative, and analgesic [73]. Carvacrol also exhibits important biological activities, such as antimicrobial (against fungi, viruses, and bacteria), immunomodulatory, antiproliferative, anti-inflammatory, and antioxidant [74]. The antibacterial activity has shown variations in the published evidence. Helander et al. [75] reported an MIC of 450 ppm for both compounds against *E. coli,* while Didry et al. [76] showed that carvacrol (MIC 125 ppm) was more effective than thymol (MIC 250 ppm) against *S. mutans.* Similarly, evaluating these compounds against *Helicobacter pylori* ATCC 43504 showed no different MICs (128 μg/mL) [77]. Meanwhile, Caballero et al. [78] reported a higher efficacy of carvacrol against *Enteroccocus* sp., with an MIC value of 100 μL/mL and 450 μL/mL for thymol. This evidence shows that both compounds could inhibit or eradicate bacterial growth; however, it has been determined that their efficacy may vary greatly depending on the tested strains.

In this context, Bautista-Hernández et al. [67] reported the terpene composition of *L. graveolens,* including α-humulene, caryophyllene oxide, and 1,8-cineole. Furthermore, this plant has quercetin O-hexoside, luteolin-glucuronide-glucoside, lithospermic acid, eriodictyol, naringenin, sakuranetin, cyrsimaritin, and chrysoeriol, among others. Leyva-López et al. [55] mentioned variations in the bioactive compositions of *O. vulgare* essential oil, depending on the location and cultivar. Thus, additional compounds to those already listed are cis-β-terpineol, β-citronellol, citronellol acetate, β-citronellal, geraniol, germacrene D-4-ol, sabinene, carvacrol methyl ether, spatulenol, rosmarinic acid, luteolin, and apigenin [72]. The chemical composition of oregano species is affected by several factors, such as environmental conditions during plant development, geographic location, soil quality, pathogens, extracted tissue, and even the collection season [55,72]. In addition, drying, extraction conditions, and sexual polymorphism also play a role. For example, in the review by Leyva-López et al. [55], it is mentioned that microwave extraction allowed for a higher yield and higher carvacrol content compared to hydrodistillation. Although similar profiles can have differences in the content of each bioactive molecule, these slight differences can cause significant changes in antibacterial and anti-biofilm potential.

The safe and ancestral use of plant-derived compounds in health and food applications is the main reason to consider these substances against antibiotic-resistant strains [79]. Regardless of the species, oregano is often used as a condiment, flavoring, and home remedy for various medical conditions, such as respiratory, stomach, and urinary diseases, and analgesics in joint conditions [72]. This versatility is related to its antioxidant, anti-inflammatory, antiproliferative, immunomodulatory, antiallergic, antipyretic, abortifacient, antiparasitic, antifungal, and antibacterial effects [67,80,81]. Knowing the human responses induced by this plant could facilitate the use of oregano terpenes to attack antibiotic resistance.

There is enough evidence to support the antibacterial activity of oregano species (Table 2); however, it is important to elucidate their mechanism of action. In both *O. vulgare* and *L. graveolens,* the sum of all their constituents could contribute to the given antibacterial capacity. However, their effect is largely attributed to their main components, carvacrol and thymol [9,55]. Some studies have investigated how these compounds carry out their activity. In the review by Sarrazin et al. [82], it is mentioned that the hydroxyl group and delocalized electrons represent a key element for this activity, in addition to the aromatic ring that makes these compounds more hydrophobic, which is related to greater interaction with the bacterial membrane [8]. In this context, deeper studies regarding the contribution of each constituent in essential oil are needed to understand their action. It is important to emphasize, as mentioned by Reyes-Jurado et al. [9], that the antibacterial capacity of these compounds resulted from a set of actions on different targets that finally led to this effect. Likewise, it is mentioned that carvacrol can cause variations in the ionic permeability of the membrane, mainly of K^+^ and H^+^, which end up leading to bacteria death. Other processes involved in the antibacterial activity of these oils are inhibiting certain enzymes and resistance mechanisms, such as efflux pumps, and eliminating biofilms and damage to the cell wall [72]. In sum, this information on the antibacterial activity of oregano allows for a better understanding of its effectiveness; however, some of these areas still require more detailed investigations. The analyzed evidence is helpful to propose that the combination of *L. graveolens* terpenes with antibiotics can show synergy and be a proper treatment against axenic and cocultured antibiotic-resistant strains, exhibiting a clear synergistic effect. Considering the diversity of terpene molecules, it can be assumed that this synergy will be greater in the essential oil–antibiotic combination. On the other hand, in the multi-species challenge, the dominant species will limit the development of surrounding species, pathogenic or beneficial.

## 6. Conclusions

Essential oils have several biological properties of great interest, especially their antibacterial activity; they can be effective even against drug-resistant bacteria. Specifically, *L. graveolens* and its terpene constituents can exert their action against bacterial viability, virulence, and antibiotic-resistance responses. The extensive evidence generated on the efficacy of *O. vulgare* or carvacrol against antibiotic-resistant bacteria allows for hypothesizing the potential efficacy of *L. graveolens* and other plant species with similar composition. In addition, further research should elucidate the mechanisms of action of plant terpenes and their mixture against bacterial viability, virulence, and resistance factors. Further, these evaluations should consider ecological interactions among different bacterial species. Finally, combining plant terpenes with conventional antibiotics is proposed to search for synergy and improved bacterial sensitivity

## Figures and Tables

**Table 1:** List of priority pathogens for research and development of new antibiotics.

Priority	Pathogens
Critical	Carbapenem-resistant *A. baumannii* Carbapenem-resistant *P. aeruginosa* Carbapenem-resistant, BLEE-producing *Enterobacteriaceae*
High	Vancomycin-resistant *E. faecium* Methicillin-resistant *S. aureus* with intermediate sensitivity and resistance to vancomycin *Helicobacter pylori* resistant to clarithromycin *Campylobacter* spp. resistant to fluoroquinolones *Salmonellae* resistant to fluoroquinolones *Neisseria gonorrhoeae* resistant to cephalosporins and fluoroquinolones
Middle	*Streptococcus pneumoniae* without penicillin sensitivity Ampicillin-resistant *Haemophilus influenzae* *Shigella* spp. resistant to fluoroquinolones

## Data Availability

Data from the reviewed articles can be found in Pubmed and Scielo.
